# Poor self-rated health is twice as common in rheumatoid arthritis: population-based evidence from the Paracelsus 10,000 study

**DOI:** 10.1007/s10238-026-02214-3

**Published:** 2026-06-20

**Authors:** Mathias Ausserwinkler, Vanessa Bartsch, Sophie Gensluckner, Bernhard Paulweber, Eugen Trinka, Patrick Langthaler, Bernhard Iglseder, Maria Flamm, Elmar Aigner, Bernhard Wernly

**Affiliations:** 1 First Department of Medicine, Landeskrankenhaus – Uniklinikum Paracelsus Medical University Salzburg, Salzburg, Austria; 2 Division of Rheumatology, Klinikum Nürnberg, Paracelsus Medical University, Nürnberg, Germany; 3 Department of Neurology, Neurointensive Care and Neurorehabilitation, Centre for Cognitive Neuroscience, Member of the European Reference Network EpiCARE, Christian Doppler University Hospital, Paracelsus Medical University Salzburg, Salzburg, Austria; 4 Neuroscience Institute, Centre for Cognitive Neuroscience, Christian Doppler University Hospital, Paracelsus Medical University, Salzburg, Austria; 5 Department of Geriatric Medicine, Christian Doppler University Hospital, Paracelsus Medical University, Salzburg, Austria; 6 Family Medicine and Preventive Medicine, Center for Public Health and Healthcare Research, Institute of General Practice, Paracelsus Medical University Salzburg, Salzburg, Austria

**Keywords:** Rheumatoid Arthritis, Self-rated health, Immunology, Paracelsus 10,000 study, Predimed score, Public Heallth

## Abstract

Self-rated health (SRH) is a simple but powerful predictor of morbidity and mortality. Its relevance in rheumatoid arthritis (RA) has not been systematically investigated in population-based cohorts. We analyzed 9,399 participants of the Paracelsus 10,000 study, including 293 (3.1%) with physician-diagnosed RA. SRH was assessed with a single-item global question. Associations of RA, sociodemographic, metabolic, and lifestyle factors with poor SRH were examined using multivariable logistic regression. Poor or very poor SRH was reported by 55% of RA participants compared with 31% of non-RA participants (*p* < 0.001). RA was independently associated with more than twofold higher odds of poor SRH (adjusted OR 2.21 (95% CI 1.61–3.02). Advanced age, metabolic syndrome, and current smoking were linked to worse SRH, whereas male sex, higher education, and higher physical activity were protective. Each one-point increase in Mediterranean diet adherence (PREDIMED score) was associated with a 4% lower odds of poor SRH (OR 0.96, 95% CI 0.94–0.99). No interaction between RA and diet adherence was observed. RA is strongly associated with poor self-rated health in the general population, after adjustment for comorbidities and lifestyle factors. Higher adherence to the Mediterranean diet and greater physical activity were linked to better perceived health, highlighting modifiable targets to improve patient-centered outcomes in RA.

**Graphical abstract:**

**Figure:**
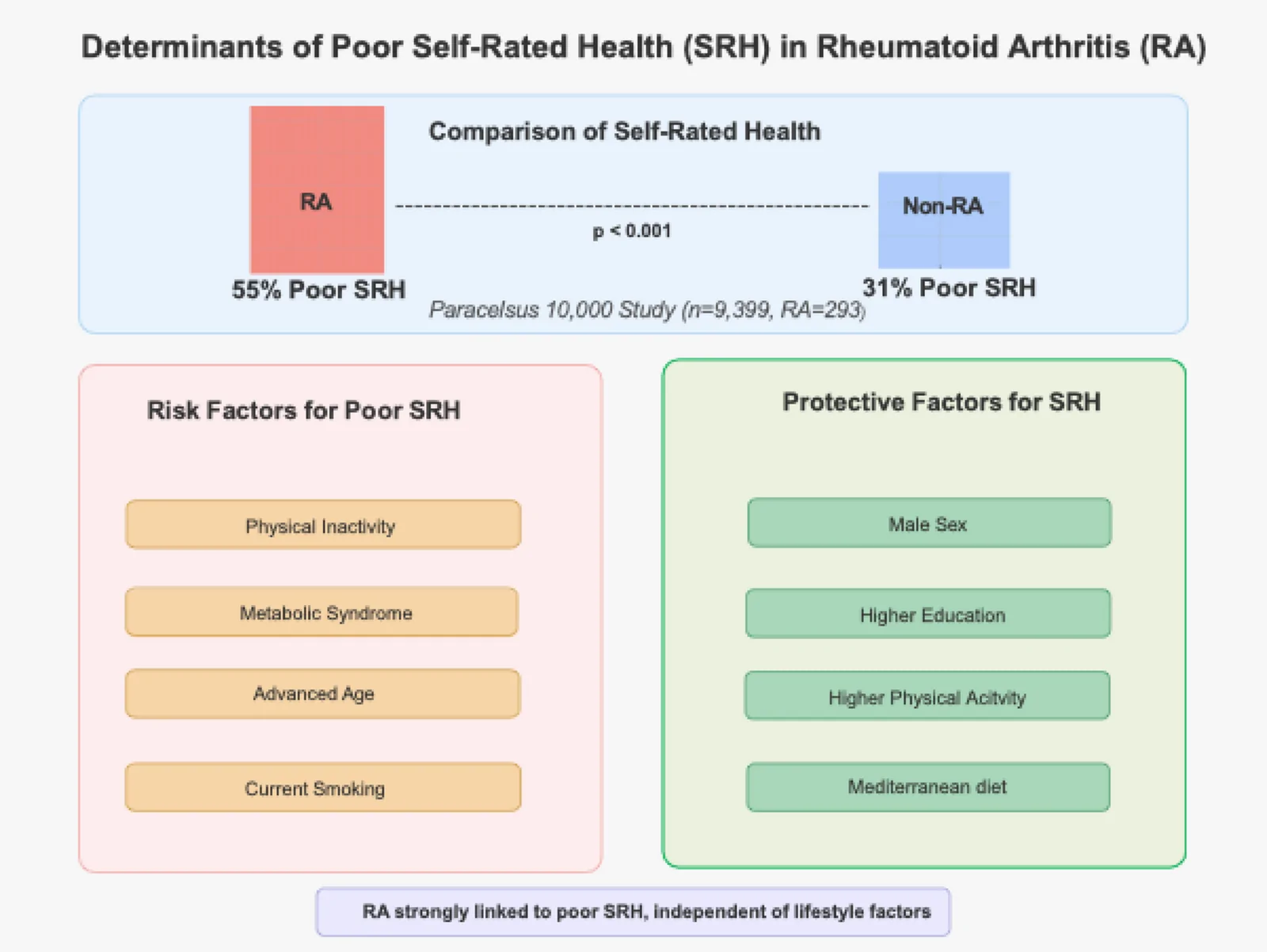

## Background

Rheumatoid Arthritis (RA) is a chronic, systemic autoimmune disease characterized by persistent synovial inflammation, extra-articular manifestations and progressive functional disability. Its global prevalence is estimated at 0.5% to 1%, disproportionately affecting women [1, 2]. Beyond musculoskeletal impairment, RA leads to a significant burden on overall health and functioning, often accompanied by comorbidities such as cardiovascular disease, infections, type 2 diabetes, depression and metabolic syndrome [3, 4]. Physical and psychological consequences of RA contribute substantially to impaired well-being and shortened life expectancy [5]. Beyond joint-related manifestations such as pain and loss of function, overall health is further compromised by fatigue and disturbed sleep [6, 7].

Self-rated health (SRH) is a simple but robust indicator of perceived general health and is an established predictor of morbidity, disability, health care utilization and all-cause mortality, independent of objective measures [8–10]. In RA, poor self-rated health may capture clinical, psychosocial, and lifestyle influences, including inactivity, obesity, and low health literacy [11, 12]. Unlike the multidimensional Short Form-36 (SF-36) General Health subscale, which captures several aspects of health status, the single-item measure offers a concise, patient-centered perspective [13, 14].

Lifestyle and nutritional factors are increasingly recognized as modifiable contributors to systemic inflammation and the development of chronic disease. Among dietary patterns, the Mediterranean diet has received particular attention for its anti-inflammatory, cardioprotective and metabolic benefits [15–17].

High adherence to this diet, typically assessed using the PREDIMED score, has been linked to improvements in both objective health outcomes and subjective well-being [18, 19]. In RA, early evidence indicates that the Mediterranean diet might influence symptoms, but its relation to perceived health could be underexplored the context of RA [20].

The Paracelsus 10,000 study is a large, population-based cohort in Salzburg, Austria, designed to investigate the interplay between lifestyle, environment and chronic disease. Here we investigate the independent relationship between RA and poor self-rated health in a population-based cohort, accounting for sociodemographic, clinical, lifestyle and dietary variables, with special attention to the Mediterranean diet [21–23].

## Methods

### Study population

This retrospective cross-sectional analysis used data from the Paracelsus 10,000 cohort, a population-based observational study conducted in Salzburg, Austria. The study enrolled adults aged 40 to 77 years between April 2013 and March 2020. Participants were randomly selected through the national resident registry, with approximately 56,600 invitation letters mailed and 10,044 individuals undergoing baseline examination. This analysis included 9,399 participants with complete data on RA status and SRH, of whom 293 had rheumatoid arthritis fulfilling the ACR/EULAR classification criteria according to their records. At study entry, an additional interview was conducted to verify rheumatoid arthritis status in accordance with the 2010 ACR/EULAR classification criteria. Disease activity scores were not collected at this time [24]. Self-rated health was assessed at baseline using a standardized questionnaire item.

### Clinical and lifestyle assessment

All participants underwent standardized evaluations, including anthropometric assesments, blood pressure measurement and fasting blood sampling. Laboratory tests included lipid profiles, HbA1c and high-sensitivity C-reactive protein (hsCRP). Lifestyle behaviors were assessed using validated questionnaires on smoking, alcohol intake, physical activity (Cardiovascular Activity Index, CAI) and dietary habits. Adherence to the Mediterranean diet was evaluated using the 14-item PREDIMED questionnaire, yielding a score between 0 and 14 (higher score indicating higher adherence). Education was classified by International Standard Classification of Education (ISCED) levels. Medical history and current medication use, including glucocorticoids and NSAIDs, were recorded through structured interviews, however individual DMARDs were not assigned or linked to specific patients. Metabolic syndrome was defined according to standard criteria as the presence of at least three of the following components: elevated abdominal circumference, elevated triglycerides, reduced HDL cholesterol, elevated blood pressure or antihypertensive treatment, and elevated fasting glucose or antidiabetic treatment.

### Outcome definition

In our study, SRH was assessed using a single standardized question: “In general, would you say your health is…?” with five response categories (“very good,” “good,” “fair,” “poor,” and “very poor”). The primary outcome was poor self-rated health, defined as participants reporting their general health as “poor” or “very poor” on the five-point scale. The primary exposure was RA status. The control group consisted of all participants without RA. Participants with chronic inflammatory bowel diseases were excluded. Secondary variables included demographic, clinical, metabolic and behavioral characteristics.

### Statistical analysis

Descriptive statistics were stratified by RA status. Continuous variables were reported as medians and interquartile ranges and compared using the Mann-Whitney U test. Categorical variables were presented as percentages and compared using chi-squared tests. Logistic regression was used to estimate odds ratios (OR) for poor self-rated health associated with RA. Three models were constructed: (I) Unadjusted, (II) adjusted for age and sex and (III) fully adjusted for age, sex, education, metabolic syndrome, physical activity, smoking status, alcohol intake and PREDIMED score. Analyses were conducted using a complete-case approach. Missing data were primarily related to lifestyle variables, particularly physical activity and dietary assessment.

Interactions between RA and all covariates were tested individually using multiplicative interaction terms. A p-value of < 0.05 was considered statistically significant. As a sensitivity analysis, ordinal logistic regression using the full five-category SRH scale as ordered outcome was performed with the same covariates as the primary binary model; the proportional odds assumption was formally assessed using the Brant test. All analyses were performed using Stata 18/BE (StataCorp, College Station, TX, USA).

### Ethics

The study was approved by the Ethics Committee of the Province of Salzburg, Austria (approval no. 415-E/1521/3-2012, approval date 12 June 2012). The approval covers the Paracelsus 10,000 cohort study and subsequent secondary analyses. The study was conducted in accordance with the Declaration of Helsinki and Austrian regulatory requirements. All participants provided written informed consent prior to enrolment.

## Results

**Fig. 1:**
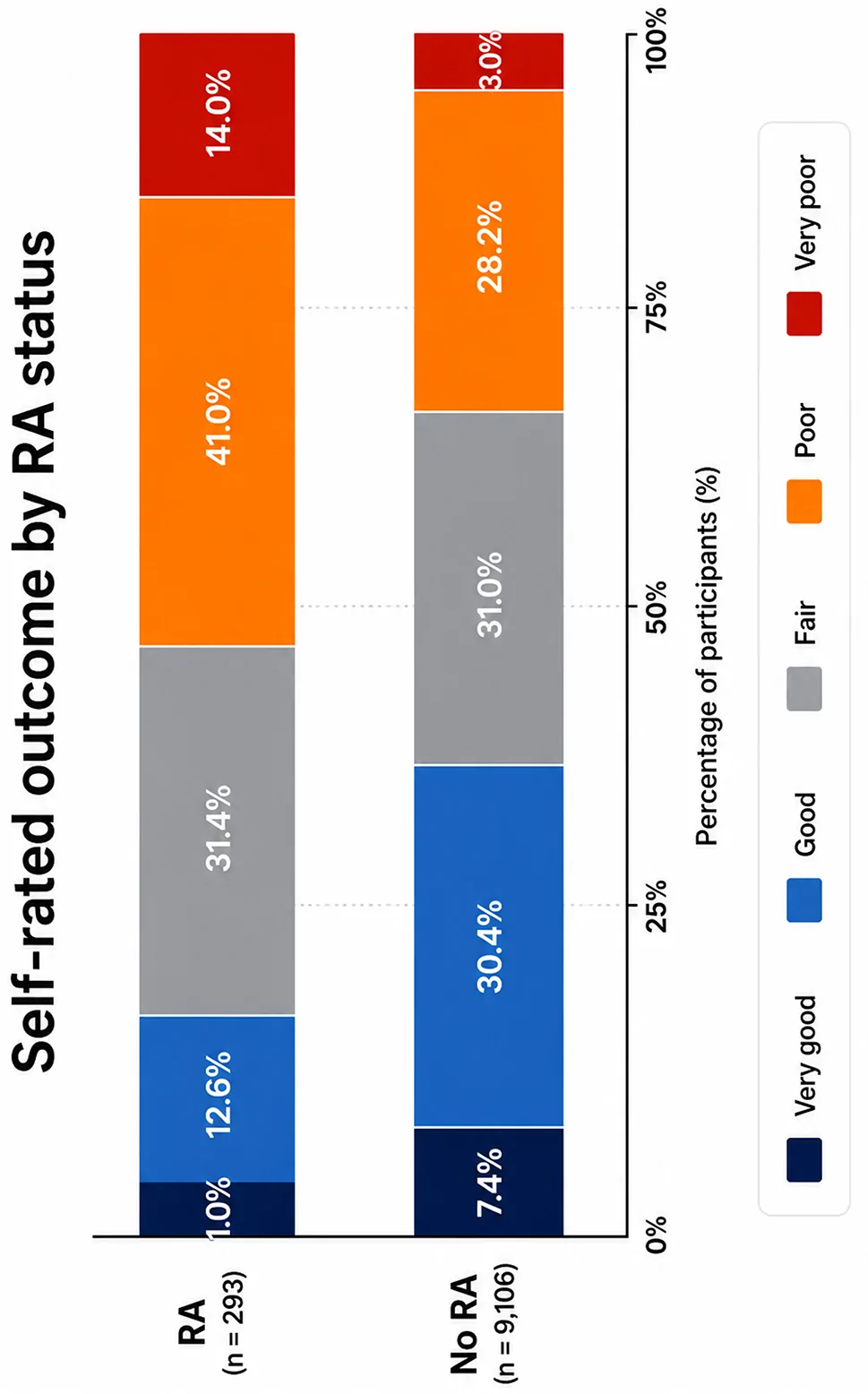
Distribution of self-rated health across the full five-point Likert scale in participants with and without rheumatoid arthritis (RA), Paracelsus 10,000 cohort. Values shown are percentages within each group

**Fig. 2:**
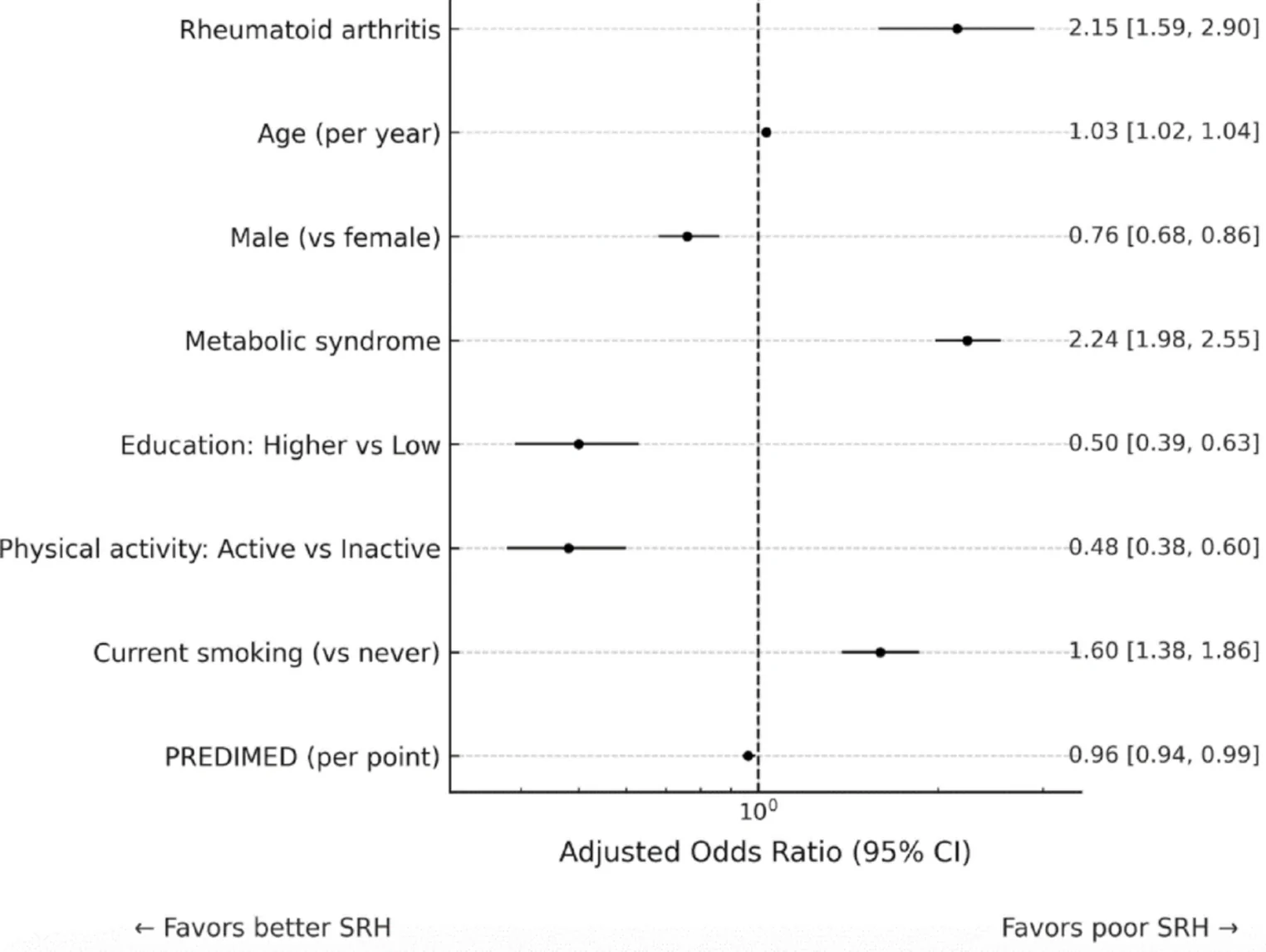
Forest plot of adjusted odds ratios (95% CI) for poor SRH from the multivariable model. Variables: RA, age, male sex, metabolic syndrome, higher vs. low education, active vs. inactive physical activity, current vs. never smoking, and PREDIMED (per point). Dashed line = OR 1; left = better SRH, right = poorer SRH. Reference groups: female, low education, inactive, never smoking

Among 9,399 participants included in the analytic sample, 293 (3.1%) were diagnosed with RA. Compared to those without RA, participants with RA were older (median age 59 vs. 55 years; *P* < 0.001) and more often female (68% vs. 51%; *p* < 0.001). Lower educational attainment was more frequent in RA (17% vs. 7% low education; *p* < 0.001). Cardiometabolic risk factors were also more prevalent, including obesity with a BMI ≥ 30 kg/m² (34% vs. 18%; *p* < 0.001), metabolic syndrome (35% vs. 25%; *P* < 0.001), type 2 diabetes (9% vs. 5%; *p* = 0.002) and hypertension (36% vs. 22%; *p* < 0.001). Median hsCRP levels were higher in RA than in non-RA participants (0.14 vs. 0.11 mg/dL; *p* < 0.001) (Table 1). Poor or very poor SRH was reported by 55% of participants with RA compared with 31% of those without RA (Fig. 1; Table 2, *p* < 0.001). In univariable analysis, RA was associated with higher odds of poor SRH (OR 2.69, 95% CI 2.13–3.40; *p* < 0.001). The association persisted after adjustment for age and sex (aOR 2.43, 95% CI 1.89–3.12; *p* < 0.001) and remained significant in the fully adjusted model that accounted for sociodemographic, metabolic and lifestyle factors (aOR 2.21 (95% CI 1.61–3.02; *p* < 0.001) (Fig. 2). Lifestyle-related factors were also associated with SRH. Greater adherence to the Mediterranean diet was independently associated with better self-rated health, with each one-point increase in the PREDIMED score corresponding to 4% lower odds of poor SRH (OR 0.96, 95% CI 0.94–0.99; *p* = 0.008). No interaction with RA status was observed (*p* = 0.256, Table 3).

**Table 1:** Differences in Characteristics of Study Participants According to Rheumatoid Arthritis Status

Characteristic	No RA (*N* = 9,106)	RA (*N* = 293)	*P* Value
Demographics			
Age, years	55 (49–61)	59 (54–64)	< 0.001
Age > 55 years	4,232 (46)	195 (67)	< 0.001
Female sex	4,648 (51)	198 (68)	< 0.001
Education level			< 0.001
Low education	663 (7)	49 (17)	
Medium education	6,231 (70)	195 (67)	
Higher education	2,071 (23)	45 (16)	
Anthropometric measures			
Body mass index, kg/m²	26 (23–29)	27 (24–31)	< 0.001
BMI ≥ 30 kg/m²	1,662 (18)	99 (34)	< 0.001
Laboratory values			
HbA1c, %	5.4 (5.3–5.6)	5.5 (5.3–5.7)	0.006
High-sensitivity CRP, mg/dL	0.11 (0.06–0.23)	0.14 (0.08–0.28)	< 0.001
Total cholesterol, mg/dL	209 (184–235)	208 (186–231)	0.91
HDL cholesterol, mg/dL	61 (51–74)	64 (52–78)	0.038
LDL cholesterol, mg/dL	140 (116–165)	134 (114–157)	0.025
Metabolic conditions			
Metabolic syndrome	2,208 (25)	101 (35)	< 0.001
Type 2 diabetes	440 (5)	26 (9)	0.002
Comorbidities			
Known hypertension	1,965 (22)	105 (36)	< 0.001
Known coronary artery disease	172 (2)	14 (5)	< 0.001
Previous stroke	172 (2)	12 (4)	0.018
Medication use			
NSAID use	605 (7)	60 (21)	< 0.001
Glucocorticoid use	29 (0.3)	22 (8)	< 0.001
Antihypertensive therapy	1,789 (20)	98 (33)	< 0.001
Statin therapy	733 (8)	30 (10)	0.18

Baseline characteristics of study participants with and without rheumatoid arthritis. Data are presented as median (interquartile range) for continuous variables and n (%) for categorical variables. P values were calculated using Mann-Whitney U test for continuous variables and chi-square test for categorical variables

Abbreviations: BMI, body mass index; CRP, C-reactive protein; HDL, high-density lipoprotein; LDL, low-density lipoprotein; NSAID, nonsteroidal anti-inflammatory drug; RA, rheumatoid arthritis

**Table 2:** Health outcomes and lifestyle behaviors according to rheumatoid arthritis status

Outcome	No RA (*N* = 9,106)	RA (*N* = 293)	*P* Value
Primary outcome			
Poor or very poor self-assessed health	2,839 (31)	161 (55)	< 0.001
Secondary outcomes			
Self-assessed health status			< 0.001
Excellent	677 (7)	3 (1)	
Good	2,768 (30)	37 (13)	
Fair	2,822 (31)	92 (31)	
Poor	2,566 (28)	120 (41)	
Very poor	273 (3)	41 (14)	
Physical activity (CAI)			0.040
Inactive	590 (7)	26 (10)	
Moderately inactive	1,999 (24)	77 (29)	
Moderately active	2,383 (29)	62 (23)	
Active	3,340 (40)	99 (38)	
Mediterranean diet adherence			
PREDIMED score (0–14)	6 (4–7)	6 (5–7)	< 0.001
Mediterranean diet adherence level			0.020
Low (0–5)	4,236 (48)	106 (39)	
Moderate (6–9)	4,276 (48)	149 (55)	
High (10–14)	372 (4)	15 (6)	
Smoking status			0.048
Never smoker	4,112 (45)	113 (39)	
Previous smoker	3,320 (36)	126 (43)	
Current smoker	1,674 (18)	54 (18)	
Alcohol consumption			
Alcohol intake, g/day	8 (2–18)	7 (2–16)	0.24
Self-reported daily alcohol intake			< 0.001
< 20/50 g/day	6,787 (89)	192 (86)	
20–30/50-60 g/day	307 (4)	21 (9)	
30–100/60-100 g/day	408 (5)	7 (3)	
> 100 g/day	104 (1)	3 (1)	

Data are presented as median (interquartile range) for continuous variables and n (%) for categorical variables. P values were calculated using Mann-Whitney U test for continuous variables and chi-square test for categorical variables

Abbreviations: CAI, Cardiovascular Activity Index; PREDIMED, Prevención con Dieta Mediterránea; RA, rheumatoid arthritis

**Table 3:** Multivariable logistic regression analysis for poor or very poor self-assessed health

Variable	Multivariable OR (95% CI)	*P* Value	Interaction OR (95% CI)	*P* Value
Rheumatoid arthritis	2.21 (1.61–3.02)	< 0.001	—	—
Age (per year)	1.03 (1.02–1.04)	< 0.001	0.99 (0.95–1.02)	0.442
Sex				
Female	Reference	—	Reference	—
Male	0.76 (0.68–0.86)	< 0.001	1.35 (0.82–2.23)	0.239
Metabolic syndrome	2.24 (1.98–2.55)	< 0.001	0.92 (0.55–1.54)	0.750
Education				
Low	Reference	—	Reference	—
Medium vs. Low	0.66 (0.53–0.83)	< 0.001	1.17 (0.59–2.30)	0.656
Higher vs. Low	0.50 (0.39–0.63)	< 0.001	0.90 (0.38–2.13)	0.802
Physical Activity				
Inactive	Reference	—	Reference	—
Moderately inactive	0.64 (0.51–0.80)	< 0.001	0.59 (0.20–1.76)	0.346
Moderately active	0.52 (0.42–0.65)	< 0.001	0.65 (0.22–1.99)	0.455
Active	0.48 (0.38–0.60)	< 0.001	0.73 (0.25–2.13)	0.565
Smoking status				
Never smoker	Reference	—	Reference	—
Previous smoker	1.07 (0.94–1.21)	0.293	0.72 (0.43–1.20)	0.206
Current smoker	1.60 (1.38–1.86)	< 0.001	1.16 (0.58–2.34)	0.677
Alcohol (g/day)	1.00 (0.996–1.001)	0.240	0.99 (0.98–1.01)	0.289
PREDIMED score	0.96 (0.94–0.99)	0.008	1.07 (0.95–1.20)	0.256

Abbreviations: CI, confidence interval; OR, odds ratio; PREDIMED, Prevención con Dieta Mediterránea. All interaction terms were non-significant (*P* > 0.05). The multivariable model included *N* = 6,845 participants

In a sensitivity analysis using ordinal logistic regression across the full five-category SRH scale, the direction, magnitude and statistical significance of all covariate effects were consistent with the primary binary model. RA remained independently associated with poorer SRH (ordinal OR 2.69, 95% CI 2.04–3.55; *p* < 0.001), and the inverse association with PREDIMED adherence was somewhat more pronounced than in the dichotomized analysis (OR 0.94, 95% CI 0.92–0.96; *p* < 0.001). These results support the robustness of our findings.

In addition, higher physical activity and higher educational attainment were independently associated with better SRH, whereas current smoking and presence of metabolic syndrome were linked to worse SRH. Alcohol intake showed no significant association with SRH (Table 3).

## Discussion

A diagnosis of RA was associated with more than twice the odds of reporting poor SRH, even after adjustment for demographic, metabolic, lifestyle and dietary factors. This finding corroborates and extends previous evidence that RA is linked not only to objective functional impairment but also to substantially diminished self-perceived health status [25–27]. An additional key observation was that greater adherence to a Mediterranean diet correlated with better SRH, independent of RA status. This association aligns with prior studies [28, 29]. However, in our study no significant interaction between RA status and Mediterranean diet adherence was observed, suggesting that the positive association with SRH applies broadly across the population. This is consistent with data indicating beneficial, but not disease-specific, effects of Mediterranean diet adherence [17, 30].

Several mechanisms may underlie the poorer SRH observed in RA. Beyond pain, fatigue and physical limitations, psychosocial factors, reduced social participation and limited health literacy likely contribute substantially. There are significant associations between RA and increased risk of depression [3]. Chronic systemic inflammation may also impair mood and well-being via cytokine-mediated pathways, while the elevated prevalence of cardiovascular and metabolic comorbidities in RA further worsens health perception [25, 26]. In addition to the association of RA with poor SRH, several lifestyle factors showed consistent and clinically meaningful relationships with perceived health. Higher levels of physical activity were strongly associated with lower odds of reporting poor SRH, in line with evidence that regular exercise improves both physical function and psychological well-being in RA and the general population. Conversely, current smoking was linked to substantially worse SRH, reflecting its known detrimental effects on inflammation, comorbidity burden, and quality of life. Alcohol consumption did not show a significant association with SRH in our analysis, which is consistent with mixed findings in previous population studies where light to moderate intake has sometimes been reported as neutral or even beneficial, whereas heavy use is harmful. Taken together, these findings highlight the importance of modifiable lifestyle behaviors in shaping self-perceived health and support integrating physical activity promotion and smoking cessation strategies into comprehensive RA management.

In this population-based analysis of the Paracelsus 10,000 cohort, the prevalence of RA (3%) was higher than the expected 1%. This difference may reflect greater participation of individuals with RA, possibly owing to a perception of limited attention within the health care system. Such differential response has been observed in other studies [23, 31–33]. In the comparison population, the proportion of 31% reporting poor or very poor self-rated health also appears strikingly high, suggesting that participants in general tended to rate their health more negatively, possibly reflecting an overall older age structure of the cohort. This also indicates the potential for selection bias, as participants may differ systematically from non-participants, which could affect the generalizability of our findings.

The key strengths of this study include its large, population-based cohort, which enhances external validity and the rigorous use of standardized, validated clinical and lifestyle assessments. The broad adjustment for demographic, metabolic, behavioral and dietary covariates minimize residual confounding and strengthens the reliability of the associations observed. Comprehensive participant characterization, supplemented by objective laboratory and anthropometric data, provides a strong foundation for interpreting SRH in the context of RA. A major limitation of this study is the lack of data on rheumatoid arthritis disease activity. Without measures such as DAS28, we could not distinguish between patients in remission and those with active disease. This limits interpretation, as poor self-rated health may reflect uncontrolled disease, pain, or functional impairment rather than broader psychosocial or lifestyle factors. Future studies including disease activity measures are needed to better clarify these relationships. Another limitation is the lack of adjustment for psychological factors such as depression and anxiety, as well as pain severity, which are known to strongly influence self-rated health, particularly in rheumatoid arthritis. The fully adjusted model was based on a reduced sample due to missing data, primarily in lifestyle variables. We used a complete-case approach, which may introduce bias if data were not missing at random. Alternative approaches such as multiple imputation was not applied and could be considered in future analyses.

Greater adherence to a Mediterranean diet was associated with better self-rated health; however, this finding should be interpreted cautiously. Given the cross-sectional design, no causal inference can be made, and reverse causation is possible, such that individuals with better perceived health may be more likely to engage in healthier dietary behaviors. In addition, the observed effect size was modest. While these findings are consistent with prior literature, they should primarily be viewed as hypothesis-generating, and longitudinal or interventional studies are needed to determine whether dietary modification can meaningfully improve perceived health. In addition, unmeasured confounding may contribute to this association. For example, individuals with higher socioeconomic status may be more likely to adhere to a Mediterranean diet, and those with better health may have greater capacity to maintain such dietary patterns. These factors cannot be fully disentangled in our cross-sectional analysis.

An important implication of our findings is the potential role of SRH as a pragmatic monitoring tool in clinical practice. Compared to multidimensional instruments such as the (SF-36) or the Health Assessment Questionnaire (HAQ), which provide granular insights but are time-consuming and therefore often underused in routine care, SRH offers a simple and rapid alternative. Despite its brevity, it strongly correlates with morbidity, mortality, and functional outcomes, making it a valuable complement rather than a replacement for established instruments. Its ease of administration could facilitate more frequent monitoring of patient well-being, particularly in resource-limited settings. Overall, SRH emerges as a powerful, patient-centered measure for understanding and enhancing outcomes in this population.

## Data Availability

No datasets were generated or analysed during the current study.
